# Genital Mycoplasmas in Placental Infections

**DOI:** 10.1155/S1064744994000244

**Published:** 1994

**Authors:** Andreas Stein, Léon Boubli, Bernard Blanc, Didier Raoult

**Affiliations:** 1 Laboratoire de Microbiologie Clinique Hôpital de la Conception Marseille France; 2 Service de Gynécologie-Obstétriqe Hôpital de la Conception Marseille France; 3 Faculté de Médecine Unité des Rickettsies 27 Boulevard Jean Moulin Marseille 13385 France

## Abstract

*Objective:* The involvement of the genital mycoplasmas *Ureaplasma urealyticum* and *Mycoplasma hominis* in complications of pregnancy has remained controversial especially because these microorganisms are frequent colonizers of the lower genital tract. Recovery of bacteria from the placenta appears to be the sole technique to represent a true infection and not vaginal contamination. Therefore, we investigated the presence of genital mycoplasmas, aerobic and anaerobic bacteria, and fungi in human placentas and evaluated their association with morbidity and mortality of pregnancy.

*Methods:* We cultured placentas from 82 women with complicated pregnancies. One hundred placentas from women with uncomplicated pregnancies were evaluated as controls. When possible, placentas were examined histologically for presence of chorioamnionitis.

*Results:* Microorganisms were recovered from 52% of the placentas of complicated pregnancies and *U. urealyticum* was the microorganism isolated most frequently from the placenta. A significant association between positive mycoplasma culture of the placenta and complication of pregnancy was found, and chorioamnionitis was positively related to isolation of mycoplasmas.

*Conclusions:* These data suggest that genital mycoplasmas are able to infect the human placenta where they can cause chorioamnionitis. This infection of the placenta by genital mycoplasmas is related to preterm birth and fatal outcome of pregnancy.


Infectious Diseases in Obstetrics and Gynecology 1:275–281 (1994)
© 1994 Wiley-Liss, Inc.

### Genital Mycoplasmas in Placental Infections

**Andreas Stein, Léon Boubli, Bernard Blanc, and Didier Raoult**

*Laboratoire de Microbiologie Clinique (A.S.) and Service de Gynécologie-Obstétrique (L.B., B.B.), Hôpital de la Conception, and Faculté de Médecine, Unité des Rickettsies (D.R.), Marseille, France*

Abstract

***Objective:*** **The involvement of the genital mycoplasmas *Ureaplasma urealyticum* and *Mycoplasma hominis* in complications of pregnancy has remained controversial especially because these microorganisms are frequent colonizers of the lower genital tract. Recovery of bacteria from the placenta appears to be the sole technique to represent a true infection and not vaginal contamination. Therefore, we investigated the presence of genital mycoplasmas, aerobic and anaerobic bacteria, and fungi in human placentas and evaluated their association with morbidity and mortality of pregnancy.**

***Methods:*** **We cultured placentas from 82 women with complicated pregnancies. One hundred placentas from women with uncomplicated pregnancies were evaluated as controls. When possible, placentas were examined histologically for presence of chorioamnionitis.**

***Results:*** **Microorganisms were recovered from 52% of the placentas of complicated pregnancies and *U. urealyticum* was the microorganism isolated most frequently from the placenta. A significant association between positive mycoplasma culture of the placenta and complication of pregnancy was found, and chorioamnionitis was positively related to isolation of mycoplasmas.**

***Conclusions:*** **These data suggest that genital mycoplasmas are able to infect the human placenta where they can cause chorioamnionitis. This infection of the placenta by genital mycoplasmas is related to preterm birth and fatal outcome of pregnancy.** © 1994 Wiley-Liss, Inc.

Key words

*Ureaplasma urealyticum*, *Mycoplasma hominis*, morbidity of pregnancy, mortality of pregnancy

The genital mycoplasmas *Mycoplasma hominis* and *Ureaplasma urealyticum* are colonizers of the female lower genital tract.[1–3] These organisms have been associated with various obstetrical complications: mortality (early spontaneous abortion, late abortion, stillbirth, neonatal death) and morbidity (premature rupture of membranes, preterm birth, low birth weight).[4] The involvement of *M. hominis* and *U. urealyticum* in these pathologies has been determined by isolation of these bacteria in the female lower and upper genital tracts (vagina, uterus, and amniotic fluid).[5–9] However, the high rate (10–60%) of genital colonization in the female population makes it difficult to appreciate the real pathogenic role of these 2 genital mycoplasmas in pregnancy.[3,4,9] A study protocol was therefore designed to correlate isolation of these microorganisms directly from the placenta with morbidity and mortality of pregnancy. A sampling of the endometrial portion of the placenta from placentas of women with complicated pregnancies was cultured for genital mycoplasmas. A control group of placentas from uncomplicated pregnancies was similarly evaluated. All placentas were also examined for other bacterial and fungal pathogens. Placentas were examined histologically when possible and the patients' charts were reviewed to evaluate specific clinical parameters.

**Address correspondence/reprint requests to Dr. Didier Raoult, Faculté de Médecine, Unité des Rickettsies, 27 Boulevard Jean Moulin, 13385 Marseille Cedex 05, France.**

Received February 2, 1994
Accepted May 12, 1994

Clinical Study

**TABLE 1. Complicated pregnancies: maternal and fetal abnormalities indicating placenta sampling (abnormality could be associated)**

| Maternal abnormalities | N | Fetal abnormalities | N |
|---|---|---|---|
| Maternal fever | 40 | Spontaneous abortion | 12 |
| Clinical evidence of infection | 10 | Stillbirth | 6 |
| Premature rupture of membranes | 14 | Preterm birth | 13 |
| Preterm labor | 18 | Low birth weight | 4 |

### SUBJECTS AND METHODS

Women were enrolled between November 1991 and November 1992 from the Unit of Gynecology and Obstetrics of the University Hospital of Marseille, France. All women admitted to the unit were screened for eligibility. Patients were selected as follows: women with documented uterine or fetal anomalies, placenta previa, abruptio placentae, or cervical cerclage were excluded from the study and women presenting with at least one maternal or fetal abnormality listed in Table 1 were included in the study. The placentas of these subjects were submitted to the laboratory for microbiological examination and culture. Placentas from women with uncomplicated pregnancies and none of the fetal or maternal abnormalities listed in Table 1 were evaluated as controls. Samples of the maternal surface of the placenta were obtained with a sterile surgical blade by slicing a 1.0 × 1.0 × 1.0 cm portion of the depth of the placenta. The samples were ground with a sterile tissue grinder and examined following Gram staining. Aerobic and anaerobic cultures for bacteria and fungi were made. Mycoplasmas were cultured in both liquid and agar media. Bacteria and fungi were identified according to the technique of Balows et al.[10] For culture of mycoplasmas, the placental tissue suspension was transferred onto a Mycofast identification strip (Groupe Stago, International Mycoplasma, Signes, France) and selective A-7 agar medium (Groupe Stago, International Mycoplasma) following the manufacturers' instructions.[11,12] Both the Mycofast strip and A-7 plates were incubated for 7 days at 37°C in a $CO_2$ atmosphere and were examined daily for the presence of *U. urealyticum* and/or *M. hominis*. Culture confirmation and numeration of mycoplasma were based on the detection of the color changes in the Mycofast identification strip and on the colonial morphology in A-7 agar. All placentas were cultured on the same day of delivery without knowledge of the subject's status. When the attending physician requested it, the placenta was examined histologically without knowledge of the results of cultures or the status of the subject. The reasons for histopathological examination of the placenta included stillbirth, neonatal death, and complications of pregnancy, labor, or delivery. Histological chorioamnionitis was defined as the demonstration of inflammatory cells, predominantly neutrophils, in the chorion and amnion.[13]

The medical records of all women were reviewed. Gestational age was estimated from the date of the mother's last menstruation, fundal height, and ultrasonography. Prematurity was defined as a gestational age of <35 weeks. Fatal outcome of pregnancy was defined as mortality due to early or late abortion, death in utero, or stillbirth.

The distribution of the culture results of the microorganisms among the study groups was examined. A $\chi^2$ test was used to evaluate the equality of proportions of positive cultures and presence of chorioamnionitis in the study groups and to correlate ratios of positive culture, chorioamnionitis, prematurity, and fatal outcome of pregnancy.

### RESULTS

In all, 82 placentas were obtained from women with complicated pregnancies and 100 placentas from women with none of the fetal or maternal abnormalities listed in Table 1 were used as controls.

The recovery of *U. urealyticum*, *M. hominis*, and other bacteria from the placenta in both groups is shown in Table 2. Microorganisms were recovered from 43 (52%) of the 82 placentas from complicated pregnancies. Twenty-two placentas (27%) contained genital mycoplasmas and 21 placentas (25%) other bacteria. Only 4 placentas (4%) of the control group grew microorganisms. The variation of the isolation rate of microorganisms among the study group and the control group was statistically significant ($P < 10^{-8}$). In the study group, *U. urealyticum* was the microorganism isolated most frequently from the placenta (24%). These data show a significant association between positive mycoplasma cultures of the placenta and complications of pregnancy ($P < 10^{-6}$).

The results of the histopathological examination of the placenta are shown in Table 3. In all, 61 of

**TABLE 2. Microorganisms recovered from placental cultures**

| | Study group (N = 82) | | Control group (N = 100) | | |
|---|---|---|---|---|---|
| | N | % | N | % | *P* |
| Genital mycoplasmas | | | | | |
| *Ureaplasma urealyticum*[a] | 20 | 24 | 1 | 1 | $<10^{-6}$ |
| *Mycoplasma hominis* | 2 | 2.4 | | | |
| Facultative bacteria | | | | | |
| Group B *Streptococcus* | 3 | 3.7 | | | |
| Viridans streptococci | 3 | 3.7 | | | |
| *Enterococcus* | 3 | 3.7 | | | |
| *Escherichia coli* | 5 | 6 | 1 | 1 | |
| *Staphylococcus aureus* | 1 | 1.2 | | | $<10^{-5}$ |
| Anaerobic bacteria | | | | | |
| *Peptostreptococcus* | 4 | 4.9 | | | |
| *Clostridium* | 1 | 1.2 | 2 | 2 | |
| Yeast | | | | | |
| *Candida albicans* | 1 | 1.2 | | | |
| Total microorganisms | 43 | 52 | 4 | 4 | $<10^{-8}$ |
| Sterile | 39 | 48 | 96 | 96 | |

[a]*U. urealyticum* was associated in 1 placenta of the study group with group B streptococci and in another placenta with *E. coli*.

**TABLE 3. Association of bacterial isolates from the placenta with histological chorioamnionitis**

| | Chorioamnionitis (placentas/placentas examined) | |
|---|---|---|
| | N | % |
| Genital mycoplasmas | | |
| *Ureaplasma urealyticum* | 11/16 | 69 |
| *Mycoplasma hominis* | — | — |
| Facultative bacteria | | |
| Group B *Streptococcus* | 2/2 | 100 |
| Viridans streptococci | 3/3 | 100 |
| *Enterococcus* | 2/3 | 66 |
| *Escherichia coli* | 4/5 | 80 |
| *Staphylococcus aureus* | 1/1 | 100 |
| Anaerobic bacteria | | |
| *Peptostreptococcus* | 1/2 | 50 |
| *Clostridium* | 1/1 | 100 |
| Total microorganisms | 25/33 | 76 |
| Sterile | 9/28 | 32 |

the 82 placentas from complicated pregnancies were examined histopathologically. Of these, 34 placentas (56%) showed histopathological chorioamnionitis. Chorioamnionitis was seen in 69% of the 16 placentas from which mycoplasmas were isolated; 32% of the sterile placentas of the study group showed inflammation. This difference in occurrence of chorioamnionitis was significant ($P < 0.002$). When comparing the sterile placentas, we noted that recovery of genital mycoplasmas or other bacteria of the placenta was significantly related to preterm birth and mortality of pregnancy (Table 4). In the group of placentas growing genital mycoplasmas, 27% were associated with preterm birth ($P < 0.03$) and 45% to fatal outcome of the pregnancy ($P < 10^{-4}$). In the group of placentas infected with bacteria other than genital mycoplasmas, 24% were associated with preterm birth ($P < 0.05$) and 33% with fatal outcome of pregnancy ($P < 0.002$). Recovery of genital mycoplasmas and other bacteria was significantly related to preterm birth and mortality of pregnancy (Table 4).

### DISCUSSION

The involvement of the genital mycoplasmas *U. urealyticum* and *M. hominis* in mortality and morbidity of pregnancy was initially suggested by isolation of these bacteria in the female lower and upper genital tracts (vagina, uterus, amniotic fluid, and endometrium)[5–9] or by serological diagnosis.[14,15] However, the role of genital mycoplasma infection in complications of pregnancy has remained controversial especially because these microorganisms frequently colonize the lower genital tract of pregnant women who are not ill and who give birth to normal, healthy infants. In fact, the high prevalence of genital colonization in the female population, which ranges from 10 to 60%, makes it difficult to accept that in some patients mycoplasmas are pathogenic, while in other there is no evidence of infection.[2] A recent review on the role of *U. urealyticum* in premature birth demonstrated that *U. urealyticum* in the lower genital tract is not directly associated with preterm birth, as preterm birth is related to a variety of risk factors for prematurity (black race, young maternal age, low educational level, low income, smoking during pregnancy, history of marijuana and/or cocaine use, and separate, single marital status). The authors conclude that these factors may be interdependent and that no consistent cause-and-effect relationship exists between the presence of *U. urealyticum* in the lower genital tract of the mother and prematurity.[9] This opinion is supported by the findings of 2 studies in which erythromycin was

**TABLE 4. Relationship of preterm birth and fatal outcome (abortion, intrauterine decease, stillbirth) of the pregnancy to bacterial infection of the placenta**

| | Placentas growing genital mycoplasmas (N = 22) | | Placentas growing bacteria other than mycoplasmas (N = 21) | | Sterile placentas (N = 39) | | Total (N = 82) | |
|---|---|---|---|---|---|---|---|---|
| | N | % | N | % | N | % | N | % |
| Preterm birth | 6 | 27 | 5 | 24 | 2 | 5 | 13 | 16 |
| Fatal outcome | 10 | 45 | 7 | 33 | 1 | 2.5 | 18 | 22 |

used to treat women with genital colonization by mycoplasmas. In these 2 studies, it was demonstrated that erythromycin treatment in women with *U. urealyticum* in the lower genital tract had virtually no impact upon low birth weight or prematurity.[16,17] As there are case reports implicating *U. urealyticum* in clinical amnionitis, investigators tried to correlate the presence of mycoplasmas in the amniotic fluid with the morbidity of pregnancy.[7,18] However, mycoplasmas are present in 50% of amniotic fluid samples from both women with intra-amniotic infection and asymptomatic control women and *U. urealyticum* does not appear to be associated with clinically evident intra-amniotic mycoplasmal infection.[19]

In fact, the recovery of bacteria from the placenta appears to be the sole technique to represent a true infection and not vaginal contamination,[9] as bacteria from the typical vaginal flora are almost never recovered from placental specimens. Furthermore, the relationship between infection of the placenta and prematurity has been shown to be independent of the duration of labor, the presence of ruptured membranes, or the duration of ruptured membranes.[20] These findings suggest that infection occurs before labor and is not a result of prolonged labor or rupture of membranes. So far, only 7 studies have examined the relationship between colonization of the placenta and outcome of pregnancy. Naessens et al.[21] cultured placentas for *U. urealyticum* but not *M. hominis* from 253 women and found no significant difference in the birth weight of infants whose placentas were colonized. Two other studies showed that the presence of *U. urealyticum* in the placenta was not related to low birth weight or prematurity.[22,23] Embree et al.[24] cultured placentas from a group of 446 "high-risk" pregnancies and 108 unselected deliveries of normal full-term infants and found an association of the isolation of *U. urealyticum* with prematurity, lower birth weight, and intrauterine growth retardation. Kundsin et al.[25] confirmed those data in a prospective study by isolating mycoplasmas more frequently from the placentas of infants who died in the perinatal period and from the placentas of those neonates admitted to the intensive case unit than from matched controls. Hillier et al.[20] related both the isolation of *U. urealyticum* alone or with other bacteria and the isolation of bacteria without *U. urealyticum* to premature delivery. In these latter 3 studies, *U. urealyticum* was isolated from the chorioamnion of 24–47% of patients who delivered low birth weight, premature infants and from 9–19% of patients who delivered at term. The frequency of isolation of *U. urealyticum* from the chorioamnion was significantly higher in those who delivered prematurely compared with those who delivered at term in these 3 studies.

As for the role of mycoplasmas in the fatal outcome of pregnancy, the 1st reports on the isolation of genital mycoplasmas from stillbirth were based on individual cases in which these organisms were isolated from fetal lungs, brain, heart, and viscera[26–30] and on 2 studies in which *U. urealyticum* was isolated more frequently from the products of early abortions and mid-trimester fetal losses than from products of induced abortions.[31,32] Embree et al.[24] isolated *U. urealyticum* more frequently from placentas of aborted fetuses than from controls. In another study of 33 perinatal death and 31 random cases of normal term deliveries, Quinn et al.[33] isolated genital mycoplasmas significantly more often from cases in which death of the fetus could not be attributed to a known anatomic or morphologic cause than from controls. Unfortunately, these authors combined their culture data with serological results and cases were considered positive if they were serologically positive even though they were culture negative.

Histological chorioamnionitis has consistently been related to morbidity and mortality of pregnancy. What causes chorioamnionitis is still a controversial subject because of the diversity of microorganisms isolated, often of low virulence, and the concept that non-specific placental inflammation may occur.[34] As related to the cause of perinatal death, no morphologic cause could be documented at autopsy of the fetus in nearly $^2/_3$ of cases and it was shown that chorioamnionitis occurs in more than 50% of these stillbirths and early neonatal deaths.[33,35] Moreover, the rates of histological chorioamnionitis are 2- to 5-fold higher in patients who deliver preterm than in those who deliver at term[20] and a direct relationship exists between the degrees of prematurity and the prevalence of histological chorioamnionitis.[13] It was shown that bacteria are recovered from the placenta of 70% of patients with histological chorioamnionitis but from only 15–45% of patients without histological chorioamnionitis.[36] *U. urealyticum* was isolated from the chorioamnion of 38–66% of patients with histological chorioamnionitis and from 13–17% of patients without histological chorioamnionitis. All of these studies which associated microbiological and histological examination of placentas revealed an increased prevalence of chorioamnionitis, spontaneous abortion, stillbirth, neonatal death, and perinatal morbidity among women with mycoplasmas isolated from the placenta compared with cases which were culture negative.[20,24,25,35] These studies suggest that *U. urealyticum* associated with chorioamnionitis may be a major factor in placental infections leading to perinatal morbidity and mortality. The aim of our study was to determine if placental infections and particularly infections with mycoplasmas represent 1 cause of preterm birth and/or fatal outcome of pregnancy. We studied the relationship between morbidity (prematurity) and mortality of pregnancy with both microbiologic and histologic findings of the placenta. A recent review of the 7 reports concerning isolation of mycoplasmas in the placenta suggests that the presence of *U. urealyticum* in the chorioamnion is only weakly associated with prematurity but strongly associated with histological chorioamnionitis.[9] Our results demonstrate an association between histological chorioamnionitis and the recovery of microorganisms from the placenta. In all, we found that in the group of complicated pregnancies 76% of the placentas growing mycoplasmas or other bacteria showed histological inflammation compared with 32% of the sterile placentas. *U. urealyticum* was the microorganism most frequently isolated from the placentas of complicated pregnancies. A lack of vaginal contamination was suggested by the absence of *Lactobacillus* isolated from the placental cultures. The presence of *U. urealyticum* in the chorioamnion appears to cause histological chorioamnionitis in the same manner as the well-known placental pathogens *Escherichia coli* and group B *Streptococcus*. Furthermore, chorioamnionitis and isolation of specific microorganisms from the placenta are associated with a significant incidence of prematurity and fatal outcome of pregnancy.

As for prematurity, pregnancies with placentas harboring genital mycoplasmas or other bacteria had significantly higher preterm birth rates (27 and 24%, respectively) than complicated pregnancies with sterile placentas (5%). As for fatal outcome of pregnancy, previous studies showed an increased incidence of chorioamnionitis and a higher isolation rate of *U. urealyticum* in perinatal deaths compared with controls, especially among those with no anatomic cause of death. In our study, chorioamnionitis was observed in 68% of pregnancies with fatal outcome. We observed a higher rate of fatal outcome in the group of pregnancies with placentas growing genital mycoplasmas (45%) than in the group growing bacteria other than mycoplasmas (33%) or in the group of sterile placentas (2.5%). These results are consistent with the report of recovery of *U. urealyticum* from discolored amniotic fluid among patients undergoing amniocentesis at 16–20 weeks gestation for genetic evaluation. In this study, patients with *U. urealyticum* in the discolored amniotic fluid delivered prematurely with evidence of severe histological chorioamnionitis and fetal *U. urealyticum* infection.[8] Furthermore, in a bovine model, ureaplasmas that were experimentally inoculated into the amniotic flud of pregnant heifers were shown to induce abortion and premature delivery associated with severe placentitis.[37,38]

In conclusion, our findings suggest that *U. ure-*

*alyticum* is emerging as an important placental pathogen. The colonization of the placenta with this microorganism, frequently associated with chorioamnionitis, constitutes a major factor in intrauterine infections associated with morbidity and mortality of pregnancy. Unfortunately, no clinically useful methods to diagnose placental infections before delivery are available. On the basis of our results, we recommend that histological and microbiological examination of the placenta be done in all cases of complicated pregnancies. Only in this manner can we better understand the pathogenesis of placental infections and develop diagnostic and therapeutic tools to improve morbidity and mortality of these pregnancies.

### REFERENCES

1. Taylor-Robinson D, McCormack WM: The genital mycoplasmas. N Engl J Med 302:1063–1067, 1980.
2. Cassell GH, Cole BC: Mycoplasmas as agents of human disease. N Engl J Med 304:80, 1981.
3. Taylor-Robinson D: *Ureaplasma urealyticum* (T-strain mycoplasma) and *Mycoplasma hominis*. In Mandell GL, Douglas RG, Bennett JE (eds): Principles and Practice of Infectious Diseases. New York: Churchill Livingstone, pp 1458–1463, 1990.
4. Cassell GH, Davis JK, Waites KB, Rudd PT, Talkington D, Crouse D, Horowitz SA: Pathogenesis and significance of urogenital mycoplasmal infections. Adv Exp Med Biol 224:93–115, 1987.
5. Romero R, Sirtori M, Oyarzun E: Infection and labor. V. Prevalence, microbiology, and clinical significance of intraamniotic infection in women with preterm labor and intact membranes. Obstet Gynecol 161:817–824, 1989.
6. Romero R, Mazor M, Oyarzun E, Sirtori M, Wu YK, Hobbins JC: Is genital colonization with *Mycoplasma hominis* or *Ureaplasma urealyticum* associated with prematurity/low birth weight? Obstet Gynecol 73:532–536, 1989.
7. Gravett MG, Hummel D, Eschenbach DA, Holmes KK: Preterm labor associated with subclinical amniotic fluid infection and with bacterial vaginosis. Obstet Gynecol 67:229–237, 1986.
8. Cassell GH, Davis RO, Waites KB: Isolation of *Mycoplasma hominis* and *Ureaplasma urealyticum* from amniotic fluid at 16–20 weeks of gestation: Potential effect on outcome of pregnancy. Sex Transm Dis 10:294, 1983.
9. Eschenbach DA: *Ureaplasma urealyticum* and premature birth. Clin Infect Dis 17:100–106, 1993.
10. Balows A, Hausler WJ, Hermann KL, Isenberg HD, Shadomy HJ: Manual of Clinical Microbiology. 5th ed. Washington, DC: American Society of Microbiology, 1991.
11. Kundsin RB, Parreno A, Poulin S: Significance of appropriate techniques and media for isolation and identification of *Ureaplasma urealyticum* from clinical specimens. J Clin Microbiol 8:445–453, 1978.
12. Shepard MC, Lunceford CD: Differential agar medium (A7) for identification of *Ureaplasma urealyticum* (human T mycoplasmas) in primary culture of clinical material. J Clin Microbiol 3:613–625, 1976.
13. Russel P: Inflammatory lesions of the human placenta. Clinical significance of acute chorioamnionitis. Am J Diagn Gynecol Obstet 1:127–137, 1979.
14. Quinn PA, Shewchuk AB, Shubern J: Serologic evidence of *Ureaplasma urealyticum* infection in women with spontaneous pregnancy loss. Am J Obstet Gynecol 145:245–250, 1983.
15. Gibbs RS, Cassell GH, Davis JK: Further studies on genital mycoplasmas in intra-amniotic infection: Blood cultures and serologic response. Am J Obstet Gynecol 154:717–726, 1986.
16. McCormack WM, Rosner BL, Lee YH, Munoz A, Charles D, Kass EH: Effect on birth weight of erythromycin treatment of pregnant women. Obstet Gynecol 69: 202–207, 1987.
17. Eschenbach DA, Nugent RP, Rao AV: A randomized placebo controlled trial of erythromycin for the treatment of *Ureaplasma urealyticum* to prevent premature delivery. Am J Obstet Gynecol 64:734–742, 1991.
18. Cassell GH, Waites KB, Gibbs RS, Davis JK: Role of *Ureaplasma urealyticum* in amnionitis. Pediatr Infect Dis 5S:341–344, 1986.
19. Blanco JD, Gibbs RS, Malherbe H: A controlled study of genital mycoplasmas in amniotic fluid from patients with intra-amniotic infection. J Infect Dis 147:650–653, 1983.
20. Hillier SL, Martius J, Krohn M, Kiviat N, Holmes KK, Eschenbach DA: A case control study of chorioamnionic infection and histologic chorioamnionitis in prematurity. N Engl J Med 319:972–978, 1988.
21. Naessens A, Foulon W, Breynaert J, Lauwers S: Postpartum bacteremia and placental colonization with genital mycoplasmas and pregnancy outcome. Am J Obstet Gynecol 160:647–650, 1989.
22. Hillier S, Krohn MA, Kiviat NB, Watts DH, Eschenbach DA: Microbiologic causes and neonatal outcomes associated with chorioamnion infection. Am J Obstet Gynecol 165:955–961, 1991.
23. Zlatnik FJ, Gelhaus TM, Benda JA, Koontz FP, Burmeister LF: Histologic chorioamnionitis, microbial infection and prematurity. Obstet Gynecol 76:355–359, 1990.
24. Embree JE, Krause VW, Embil JA, McDonald S: Placental infection with *Mycoplasma hominis* and *Ureaplasma urealyticum*, clinical correlation. Obstet Gynecol 56:475–481, 1980.
25. Kundsin RB, Driscoll SG, Monson RR, Yeh C, Biano SA, Cochran WD: Association of *Ureaplasma urealyticum* in the placenta with perinatal morbidity and mortality. N Engl J Med 310:941–945, 1984.
26. Quinn PA, Gilan JE, Markstad T: Intrauterine infection with *Ureaplasma urealyticum* as a cause of fatal neonatal pneumonia. Pediatr Infect Dis 4:538, 1985.

27. Brunnell PA, Dische MR, Walker M: Mycoplasma amnionitis and respiratory distress syndrome. JAMA 207: 2097, 1969.
28. Romano N, Romano F, Carollo F: T-strains of mycoplasmas in broncopneumonic lungs of an aborted fetus. N Engl J Med 285:950, 1971.
29. Pease P, Rogers KB, Cole BC: A cytopathogenic strain of *Mycoplasma hominis* type I isolated from the lung of a stillborn infant. J Pathol Bacteriol 94:460, 1967.
30. Tafari N, Ross S, Naeye RL: Mycoplasma T strains and perinatal death. Lancet 1:108, 1976.
31. Sompolinsky D, Solomon F, Eikina L: Infections with mycoplasma and bacteria in induced mid-trimester abortion and fetal loss. Am J Obstet Gynecol 121:610, 1975.
32. Caspi E, Solomon F, Sompolinsky D: Early abortion and *Mycoplasma* infection. Isr J Med Sci 8:122, 1972.
33. Quinn PA, Butany J, Chipman M: A prospective study of microbial infection in stillbirth and early neonatal death. Am J Obstet Gynecol 151:238–249, 1985.
34. Pankuch GA, Appelbaum PC, Lornz RP, Botti JJ, Schachter L, Nave RL: Placental microbiology and histology and the pathogenesis of chorioamnionitis. Obstet Gynecol 64:802–806, 1984.
35. Quinn PA, Butany J, Taylor J, Hannah W: Chorioamnionitis: Its association with pregnancy outcome and microbial infection. Am J Obstet Gynecol 156:379–387, 1987.
36. Gibbs RS, Romero R, Hillier SL, Eschenbach DA, Sweet RL: A review of premature birth and subclinical infection. Am J Obstet Gynecol 166:1515–1518, 1992.
37. Miller RB, Ruhnke RL, Doig BF: The effects of *Ureaplasma diversum* inoculated into the amniotic cavity in cows. Theriogenology 20:367, 1983.
38. Ruhnke RL, Palmer NC, Doig PA: Bovine abortion and neonatal death associated with *Ureaplasma diversum*. Theriogenology 21:295, 1984.
